# Identification of transcription factors that regulate placental sFLT1 expression

**DOI:** 10.1093/molehr/gaaf031

**Published:** 2025-07-09

**Authors:** Qing Yong, Carin van der Keur, Jacqueline D H Anholts, Hanneke Kapsenberg, Hailiang Mei, Jan A Bruijn, Michael Eikmans, Hans J Baelde

**Affiliations:** Department of Pathology, Leiden University Medical Centre, Leiden, The Netherlands; Department of Immunology, Leiden University Medical Center, Leiden, The Netherlands; Department of Immunology, Leiden University Medical Center, Leiden, The Netherlands; Department of Immunology, Leiden University Medical Center, Leiden, The Netherlands; Sequencing Analysis Support Core, Leiden University Medical Center, Leiden, The Netherlands; Department of Pathology, Leiden University Medical Centre, Leiden, The Netherlands; Department of Immunology, Leiden University Medical Center, Leiden, The Netherlands; Department of Pathology, Leiden University Medical Centre, Leiden, The Netherlands

**Keywords:** soluble FMS-like tyrosine kinase 1, transcription factor, trophoblast, inflammation, early pregnancy, FOXM1, CEBPB

## Abstract

Increased soluble FMS-like tyrosine kinase 1 (sFLT1) levels have been associated with preeclampsia, chronic kidney diseases, and kidney transplant rejection. However, lower levels of sFLT1 exhibit beneficial properties in various processes, such as the organization of the actin cytoskeleton in podocytes and immune regulation in healthy pregnancy. Therefore, understanding the transcriptional regulation of sFLT-1 and preserving appropriate expression levels are critical for effective treatment of preeclampsia and other diseases. Cytotrophoblasts (CTBs) were isolated from three first-trimester placentas and differentiated into extravillous trophoblasts (EVTs) for 6 days. RNA was extracted at different time points and used for RNA sequencing. Differentially expressed genes (DEGs) and transcription factors (DETFs) were analyzed. Transcription factor (TF) enrichment analysis and pathway analysis were performed on DEGs screened from EVTs and CTBs. TF inhibitors were added to primary CTBs directly or during CTB to EVT differentiation to confirm the regulatory effect of TFs on sFLT1 expression. In total, 197 TFs were differentially expressed between CTBs and EVTs, among which 15 DETFs (EPAS1, ETS1, TBX3, CEBPB, FLI1, TEAD4, GATA4, TBX2, LMX1B, ARNT, FOXM1, ERF, PRDM1, TFAP2A, and NR2F2) that potentially regulate sFLT1 expression were predicted by ChEA3 and KnockTF software. The mRNA levels of 15 DETFs were validated upon CTBs differentiation into both EVTs and syncytiotrophoblasts. The regulatory effects of FOXM1 and CEBPB were confirmed *in vitro* experiments, and their expression patterns were validated during CTBs differentiation into EVTs and in first-trimester placentas. Pathway analysis showed that FLT1 was involved in P13K-Akt, Rap1, MAPK, Ras, and HIF-1 signaling pathways, focal adhesion, and cytokine–cytokine receptor interaction. Protein–protein interaction analysis showed that FLT4, PDGFB, TGFB1, IL6R, TNFRSF1B, CSF1R, and TGFB2 interact with FLT1. The identified TFs can serve as therapeutic targets in preeclampsia to keep the sFLT1 levels within appropriate limits.

## Introduction

Soluble FMS-like tyrosine kinase 1 (sFLT1) is a splice variant of vascular endothelial growth factor receptor-1 (VEGFR1/FLT1). Compared to the full-length membrane receptor FLT1, sFLT1 lacks the seventh Ig-like domain, as well as the transmembrane and tyrosine kinase regions (Jena *et al.*, 2020). As a result, it plays an anti-angiogenic role by acting as a VEGF decoy receptor through capturing circulating VEGF and placental growth factor (PIGF) and by forming dimers with VEGF receptors in a non-signaling manner (Cudmore *et al.*, 2012).

Soluble FLT1 has been associated with preeclampsia (PE), which is a severe complication affecting 5–7% of pregnancies worldwide with high morbidity and mortality (Rana *et al.*, 2019). PE is defined as new-onset hypertension accompanied by proteinuria and/or other maternal organ dysfunction and/or uteroplacental dysfunction after 20 weeks of gestation (Brown *et al.*, 2018). Although the pathogenesis of PE is not fully understood, excess sFLT1 is believed to be the primary cause of maternal vascular symptoms through its antagonistic effect on VEGF and PIGF (Maynard *et al.*, 2003). Similarly, in patients with preterm PE, extracorporeal removal of circulating sFLT1 alleviates proteinuria and hypertension and potentially prolongs pregnancy duration (Thadhani *et al.*, 2011). Moreover, the disruptive effects of overexpressing sFLT1 on spiral arterial remodeling and metabolic alterations have been demonstrated in PE mouse models (Sato *et al.*, 2020; Vogtmann *et al.*, 2021). In addition, increased serum sFLT1 has been observed and linked to renal impairment in the context of chronic kidney diseases and kidney transplantation (Wewers *et al.*, 2021).

In contrast to these detrimental effects, sFLT1 exhibits beneficial properties in various processes. For instance, physiological levels of sFLT1 play a crucial role in regulating the actin cytoskeleton of podocytes (Jin *et al.*, 2012). Moreover, treatment with certain doses of sFLT1 reduces systemic inflammation and influx of macrophages at the site of damage in preclinical models of inflammatory disease (Biscetti *et al.*, 2016; Bus *et al.*, 2017; van Aanhold *et al.*, 2020). In line with this, the level of sFLT1 in the maternal circulation dramatically increases in normal pregnancies compared to the non-pregnant state (Levine *et al.*, 2004), and the modulatory effect of sFLT1 in trophoblast invasion during healthy pregnancy has been supported by a previous study (McMahon *et al.*, 2014). Furthermore, the protective upregulation of sFLT1 in response to VEGF overexpression is essential for maintaining vascular integrity in the placenta (Fan *et al.*, 2014).

Given the complex and diverse role of sFLT1 in pregnancy and other pathophysiological conditions, understanding the transcriptional regulation of sFLT-1 and preserving appropriate expression levels are critical for effective treatment of PE and other diseases. During pregnancy, trophoblast cells serve as the main source of sFLT1, which may enter the maternal circulation (Clark *et al.*, 1998). In a previous study of early healthy pregnancy, we reported that sFLT1 is predominantly produced by extravillous trophoblasts (EVTs) and syncytiotrophoblasts (STBs), but not by cytotrophoblasts (CTBs) (Yong *et al.*, 2023). Here, we set out to identify transcription factors (TFs) that potentially regulate sFLT1 expression upon differentiation of human primary trophoblasts into EVTs or STBs, and we validated the regulatory effects of TFs *in vitro*. In addition, we investigated the pathways of differentiation in which sFLT1 is involved.

## Materials and methods

### Human tissue

First-trimester placentas of three donors were used to collect tissue for immunohistochemistry and to isolate primary trophoblasts as previously described (Eikmans *et al.*, 2022) using the trophoblast stem cells derivation methods developed by Okae *et al.* (2018). Hereafter, we refer to these primary trophoblasts as CTBs. The donors terminated pregnancy at 5-, 6-, or 12-weeks gestation due to social reasons.

All samples were collected and handled anonymously, in accordance with Dutch national ethics guidelines and the Code of Conduct regarding the Proper Secondary Use of Human Tissue.

### Cell culture

Since sFLT1 is up-regulated in EVTs and STBs, isolated CTBs from first-trimester placentas were differentiated into EVTs and STBs *in vitro* (Okae *et al.*, 2018). CTBs were cultured on plates coated with 5 µg/ml of collagen IV (Sigma-Aldrich, Burlington, MA, USA) in CTB medium, which was DMEM/F12 (Gibco, Waltham, MA, USA) supplemented with 0.05 mM 2-mercaptoethanol (Gibco), 0.2% v/v fetal bovine serum (Gibco), 1% v/v penicillin/streptomycin (p/s) (Gibco), 0.3% w/v bovine serum albumin (BSA) (Fujifilm, Tokyo, Japan), 0.5% v/v Knockout Serum Replacement (KSR) (Gibco), 1% v/v Insulin–Transferrin–Selenium (ITS)-X (Gibco), 1.5 µg/ml of L-ascorbic acid (Sigma-Aldrich), 50 ng/ml of epithelial growth factor (EGF; Miltenyi Biotec, Bergisch Gladbach, Germany), 2 µM CHIR-99021 (StemCell Technologies, Vancouver, BC, Canada), 0.5 µM A83-01 (StemCell Technologies), 1 µM SB431542 (StemCell Technologies), 0.8 mM Valproic acid (Fujifilm), and 5 µM Y-27632 (StemCell Technologies). Plates were incubated with collagen IV for 90 min at 37°C. Then, 0.5 × 10^6^ CTBs were plated in 2 ml of CTB medium. During culturing, the medium was refreshed every 2–3 days.

For EVT differentiation, 0.75 × 10^5^ CTBs were transferred to plates coated with 1 µg/ml of collagen IV and incubated in 2 ml of EVT medium, which included DMEM/F-12 medium supplemented with 0.1 mM 2-mercaptoethanol, 1% v/v p/s, 0.3% w/v BSA, 1% v/v ITS-X, 7.5 µM A 83-01, 2.5 µM Y-27632, 100 ng/ml of Neuregulin 1 (NRG1) (Cell Signaling Technology, Danvers, MA, USA), and 4% v/v KSR. Finally, 2% v/v Matrigel^®^ (Merck, Darmstadt, Germany) was added to cells with EVT medium. After 3 days, the medium was replaced with the same content but without NRG1 and with a Matrigel concentration of 0.5% v/v. On Day 6, cells were harvested for mRNA measurement and immunochemistry.

For STB differentiation, 1 × 10^5^ CTBs were transferred to plates coated with 2.5 µg/ml of collagen IV and incubated in 2 ml of STB medium, which contained DMEM/F-12 supplemented with 0.1 mM 2-mercaptoethanol, 1% v/v p/s, 0.3% w/v BSA, 1% v/v ITS-X, 2.5 µM Y-27632, 2 µM forskolin (StemCell Technologies), and 4% v/v KSR. After 3 days, the medium was replaced with the same content. At 6 days, the cells were harvested for further analysis.

To study the effect of FOXM1 on sFLT1 expression, 3.5 × 10^5^ CTBs per well with CTB medium were plated in a six-well plate. One day after the cells reached confluency, they were incubated with 5, 10, 20, or 40 µM FOXM1 inhibitor (FDI-6) (Sigma-Aldrich, Burlington, MA, USA) for 24 h. Then, the cells were harvested for further measurements.

To study the effect of CEBPB inhibition on sFLT1 expression during EVT differentiation, 0.75 × 10^5^ CTBs per well with EVT medium were plated in a six-well plate. After 3 days, medium for EVTs was changed, and 0.5, 1, or 2 µM CEPBP inhibitor (Helenalin acetate) (MedChemExpress, Monmouth junction, NJ, USA) was added. On Day 5, all samples were harvested for gene expression analysis.

To assess the cytotoxicity of Helenalin acetate on trophoblasts, a cell viability assay was performed with PrestoBlue (Invitrogen, Waltham, MA, USA); 1 × 10^4^ CTBs per well with CTB medium were plated in a 96-well plate. One day after the cells reached confluence, they were incubated with different concentrations of Helenalin acetate (0, 0.5, 1, 2, or 4 µM) for 24 h. Then, the culture medium was discarded, and cells were washed twice with a cold phosphate buffer solution. Medium mixed in a 1:9 ratio of PrestoBlue to fresh medium was added to the cells and incubated at 37°C for 1 h. Absorption at 538 nm with a reference wavelength of 600 nm was measured using a spectrophotometer (Tecan, Zürich, Switzerland).

### RNA extraction and quantitative PCR

To quantify changes in gene expression, total RNA was extracted from cells using TRIzol reagent (Thermo Fisher Scientific, Waltham, MA, USA) and converted to cDNA. Quantitative real-time PCR was performed using IQ SYBR Green Supermix (Bio-Rad, Hercules, CA, USA) on a Bio-Rad CFX real-time system. Gene expression levels were normalized to the housekeeping gene *GAPDH*, *TBP*, and *HPRT*. The human primers listed in Table 1 were used.

**Table 1.** Primer sequences for real-time PCR analysis.

Target	Forward (5′–3′)	Reverse (5′–3′)
*GAPDH*	CGACCACTTTGTCAAGCTCA	AGGGGTCTACATGGCAACTG
*sFLT1*	CGAGCCTCAGATCACTTGGT	CGATGACGATGGTGACGTT
*EPAS1*	CGGAGGTGTTCTATGAGCTGG	AGCTTGTGTGTTCGCAGGAA
*TBX3*	AAAGGTTTCCGGGACACTGG	GCTGCTTGTTCACTGGAGGA
*CEBPB*	AGAAGACCGTGGACAAGCAC	GCTTGAACAAGTTCCGCAGG
*FLI1*	AGGCTGTAACCGGGTCAATG	ACGCTGAGTCAAAGAGGGAC
*TEAD4*	TCCTTGGAACTGGCTTAGCG	TCGTTGGAGGTAATGGTGCC
*TBX2*	CAGTGGATGGCTAAGCCTGT	TTCAGGATGTCGTTGGCTCG
*LMX1B*	CTGCTGTGCAAGGGTGACTA	CGGCTTCATGTCCCCATCTT
*ARNT*	GGAATGGACTTGGCTCTGTAA	GTCATCATCTGGGAGGAAAC
*FOXM1*	ACGTGGATTGAGGACCACTT	GCAATTGTGGAGACCCTGG
*PRDM1*	CCCGGAGAGCTGACAATGAT	GTCCTTTCCTTTGGAGGGGT
*TFAP2A*	AGAGGGGCATATCCGTTCAC	CTCGCAGTCCTCGTACTTGA
*NR2F2*	GCCCGGGTAGCGACAAG	CACGTGAACTGGCCGTAGTG

### RNA sequencing and differentially expressed genes identification

CTBs and EVTs on Days 3 and 6 were collected and used for RNA extraction as described above. The quality and integrity of RNA were controlled with an Agilent 2100 Bioanalyzer: RNA Integrity Number (RIN) values were >9.5.

RNA-Seq FASTQ files were processed using the open-source BIOWDL RNAseq pipeline v5.0.0 developed in Leiden University Medical Centre (LUMC). This pipeline performs FASTQ preprocessing (including quality control, quality trimming, and adapter clipping), RNA-Seq alignment, and read expression quantification. FastQC was used for checking raw read QC. Adapter clipping was performed using Cutadapt (v2.10) with default settings. RNA-Seq reads alignment was performed using STAR (v2.7.5a) on the GRCh38 human reference genome. The gene read quantification was performed using HTSeq-count (v0.12.4) with the setting ‘–stranded=yes’. The gene annotation used for quantification was Ensembl version 104. Using the gene read count matrix, counts per million (CPM) was calculated per sample on all annotated genes. Genes with a CPM higher than 1 in at least 25% of all samples were kept for downstream analysis. For the differential gene expression analysis, the dgeAnalysis R-shiny application was used. EdgeR (v3.28.1) with TMM normalization was used to perform differential gene expression analysis. The Benjamin and Hochberg false discovery rate (FDR) was computed to adjust *P*-values obtained for each differentially expressed gene (DEG). |log2FC| > 1 and adjusted *P*-value <0.05 were regarded as the statistically significant threshold for DEGs. Volcano plots were drawn to visualize DEGs using the ‘tidyverse’ package in R 4.3.1. Heatmaps were created with the ‘pheatmap’ package by taking the top 500 most variable genes across all samples.

### TF enrichment analysis

Human TFs database (version 1.01) (https://humantfs.ccbr.utoronto.ca/) was used for the identification of differentially expressed transcription factors (DETFs).

TFs were predicted on DEGs selected from group EVTd6 and CTB using the web-based enrichment analysis tool, ChEA3 (https://maayanlab.cloud/chea3/) and KnockTF 2.0 (https://bio.liclab.net/KnockTF/index.php). JASPAR 2024 database (https://jaspar.elixir.no/) was used to confirm the presence of binding sites for differentially expressed TFs at the promoter of the *FLT1* gene.

### Signal pathway enrichment analysis

Kyoto Encyclopedia of Genes and Genomes (KEGG) enrichment analysis was performed on DEGs screened from the groups EVTd6 and CTB using DAVID 2021 online software (https://david.ncifcrf.gov/) to explore the biological pathways of DEGs. Analysis was statistically significant at *P* < 0.05 and enriched genes >5. Bubble charts were plotted by using the ‘ggplot2’ package of R software to visualize analysis.

### Gene set enrichment analysis

To better understand molecular signaling pathways involved in sFLT1, we performed gene set enrichment analysis (GSEA) software (version 4.3.2) using all detected genes. The ‘c2.cp.kegg_legacy. v2023.2.Hs. symbols’ downloaded from the GSEA molecular signature database was selected as the reference gene set. |Normalized enrichment score (NES)| > 1, FDR <0.25 and *P* < 0.05 were considered as the cutoff criteria. Subsequently, protein–protein interaction (PPI) network was established with The STRING online search tool (https://string-db.org/).

### Immunochemistry

We performed multiplexed immunohistochemical consecutive stainings on the same slides following a previous study (Remark *et al.*, 2016). Both formalin-fixed cell pellets and placental tissues were embedded in paraffin, and sections (4 µm thickness) were cut. After deparaffinization in xylene and rehydration in decreasing concentrations of ethanol (100, 70, 50%, and distilled water), rehydrated tissue sections were incubated in Tris/EDTA buffer for antigen retrieval at 95°C for 1 h. Hydrogen peroxide solution (Merck, Darmstadt, Germany) and serum-free protein block (Dako, Glostrup, Denmark) were used for blocking. Samples were incubated with rabbit anti-human FOXM1 (1:200, ABE1000, Merck, Darmstadt, Germany) antibody for 1 h at room temperature. The ready-to-use EnVision—horseradish peroxidase (HRP) anti-rabbit (Dako Cytomation, Glostrup, Denmark) was used with NovaRED (Vector Laboratories, Newark, CA, USA) as a chromogen. Tissue sections were then mounted with glycergel mounting medium (Dako, Glostrup, Denmark). After scanning, slide coverslips were removed in hot (∼56°C) water, and tissue sections were destained in organic solvent (50% ethanol, 2 min; 70% ethanol + 1% HCl, 5 min; 100% ethanol, 5 min; 70% ethanol, 2 min; 50% ethanol, 2 min). Then, the slides were washed in distilled water for 5 min and subjected to the next round of staining. Antigen retrieval was performed using Tris/EDTA or citrate buffer at 95°C for 30 min. After blocking with hydrogen peroxide and serum-free protein, Fab fragment (Jackson ImmunoResearch, West Grove, PA, USA) was used to block the primary antibody used previously. Then, the sections were sequentially stained with either mouse anti-human CEBPB (1:1000, 66649-1-Ig, Proteintech, Manchester, UK), rabbit anti-human FLT1 (1:100, MA5-50127, Invitrogen, Waltham, MA, USA), mouse anti-human HLA-G (1:800, 11-436-C100, ExBio, Nad Safinou, Vestec, Czech Republic), or rabbit anti-human Chorionic Gonadotropin (HCG, 1:80 000, A0231, Agilent, Santa Clara, CA, USA). The EnVision—HRP anti-mouse or anti-rabbit antibodies were used as secondary antibodies and visualized using NovaRED.

### Flow cytometry

To quantify protein levels of FOXM1, CEBPB, and sFLT1/FLT1, flow cytometry was used. Cells were incubated for 30 min in Fixation/Permeabilization diluent (Thermo Fisher Scientific, 00-5123-43 and 00-5223, Waltham, MA, USA) on ice, followed by washing with Permeabilization Buffer (Thermo Fisher Scientific, 00-8333). Cells were subsequently incubated with either mouse anti-human CEBPB (1:250), rabbit anti-human FOXM1 (1:200), or rabbit anti-human FLT1 for 30 min on ice, which were detected using either Alexa Fluor 546 anti-rabbit or mouse (1:400, Thermo Fisher) or Alexa Fluor 647 anti-rabbit (1:400, Thermo Fisher). LIVE/DEAD™ Fixable Near IR (780) Viability Kit (Thermo Fisher Scientific, L34994) was applied to exclude dead cells. Normal mouse or rabbit serum (Dako, Glostrup, Denmark) was used as a negative control. Analysis was performed using a BD LSRFortessa (BD Biosciences, San Jose, CA, USA).

### Analysis of secreted sFLT1

Supernatants of trophoblast cultures were analyzed for the presence of secreted sFLT1 using the Luminex assay (Bio-Techne, LXSAHM-02, Minneapolis, MN, USA) following the manufacturer’s instructions. The assay could not distinguish between sFLT1 and FLT1, but we used cell supernatants for measurements. Therefore, we assume we measured sFLT1 levels. Samples were analyzed using a Luminex 200 with Bio-plex Manager 6.2 Software (Bio-Rad Laboratories, Hercules, CA, USA). All samples were measured in duplicate.

### Statistical analyses

Summary data are presented as means ± SD from three independent experiments. One-way ANOVA was performed for multigroup comparisons, followed by the LSD multiple comparison test for subgroup comparison in case the overall *F*-test was significant. Paired *t*-test was used to quantify the protein expression of TFs with flow cytometry. To analyze the effect of TF inhibition on sFLT1 expression, paired *t*-test or a linear regression model was used. All normalized gene expression data were log-transformed, and an alpha level of 0.05 was taken to assess statistical significance.

## Results

### Identification of DEGs

**Figure 1.**
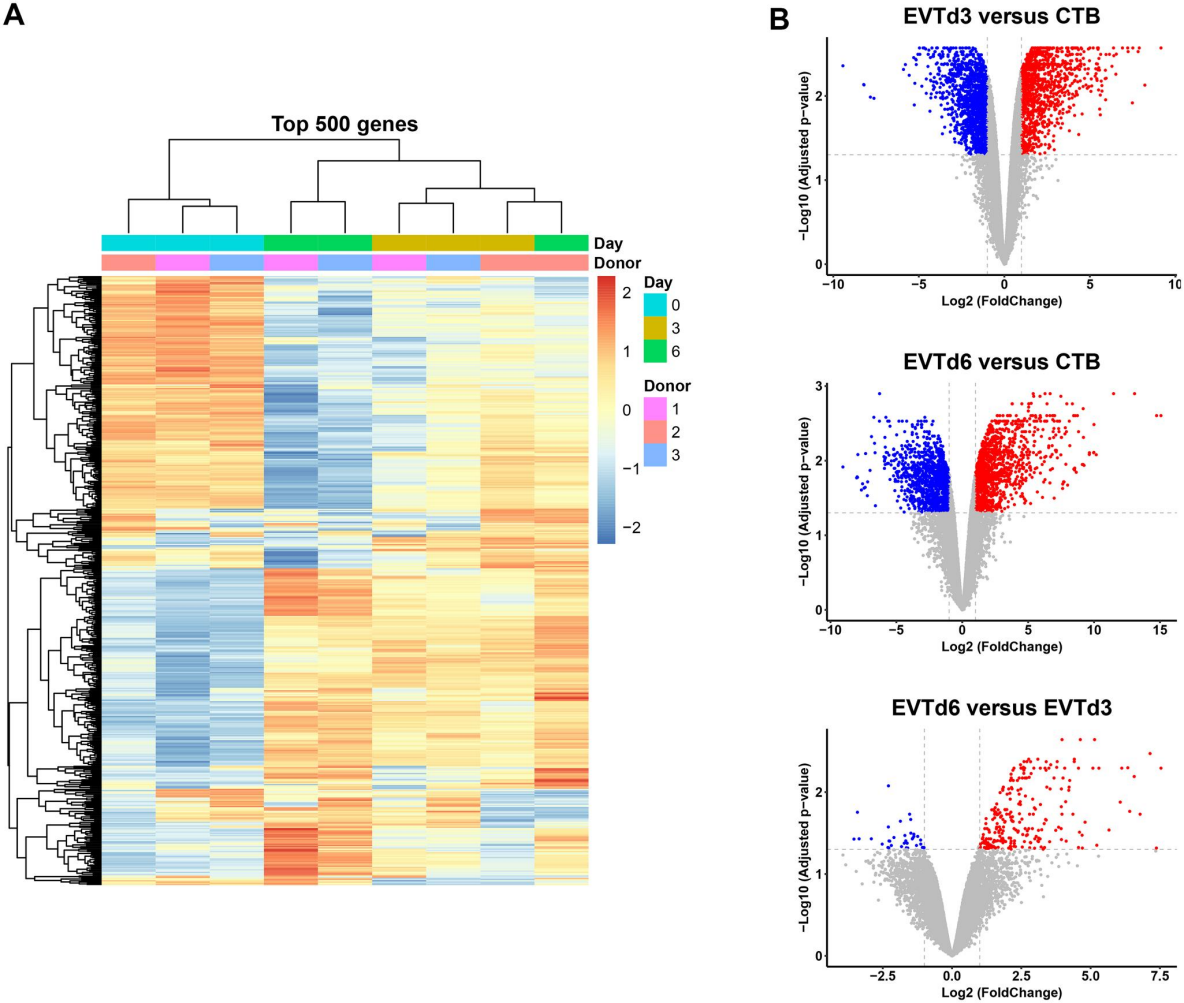
**Identification of differentially expressed genes in cultured trophoblasts.** Cytotrophoblasts (CTBs) were isolated from first-trimester placentas of three donors and differentiated into extravillous trophoblasts (EVTs). These were collected on Days 3 and 6 separately. (**A**) Hierarchical clustering of the top 500 of most variable genes across all samples. (**B**) Volcano plots of the differentially expressed genes between groups (CTB, EVTd3, EVTd6). All the genes were plotted, and each data point represents a gene. The log2 fold change of each gene is represented on the *x*-axis and the log10 of its adjusted *P*-value is on the *y*-axis. Genes with an adjusted *P*-value <0.05 and fold change more than two times are indicated by red (up-regulated genes) and blue (down-regulated genes) dots.

To acquire an overview of alterations and to identify DEGs during differentiation of CTBs into EVTs, we isolated CTBs from first-trimester placentas and differentiated them into EVTs. RNA from these cells was subjected to sequencing analysis. We first generated a bi-clustering heatmap to depict the gene expression profile using the top 500 most variable genes across all samples (Fig. 1A). CTBs from three distinct placentas clustered together and showed similar gene expression patterns. The gene expression profiles were highly altered upon differentiation of CTBs into EVTs.

Next, we performed differential gene expression analysis by comparing the following groups: EVTd3 versus CTB, EVTd6 versus CTB, and EVTd6 versus EVTd3. Differentially up- and down-regulated genes per comparison are shown in the volcano plots (Fig. 1B). Compared to CTBs, EVTs on Day 3 had 1258 significantly up-regulated genes and 1433 down-regulated genes (Supplementary Table S1). For EVTs on Day 6, 1380 up-regulated and 1253 down-regulated genes were identified when compared with CTBs (Supplementary Table S2). The gene expression profile of EVT on Day 6 was only slightly different from that of EVT on Day 3, with only 256 genes up-regulated and 37 genes down-regulated significantly (Supplementary Table S3). As expected, FLT1 was 6.3 times and 35.5 times up-regulated on Day 3 and 6 of EVT differentiation, respectively, when compared to Day 0 (CTBs).

### Prediction of differentially expressed TFs associated with FLT1

Since FLT1 had the highest expression on Day 6 during differentiation, we performed TF enrichment analysis on group EVTd6 and CTB. First, we identified DETFs with 90 up-regulated and 107 down-regulated from these two groups (Supplementary Table S4). Then DEGs screened from comparison of EVTd6 and CTB were subjected to the online tool ‘ChEA3’ (Keenan *et al.*, 2019): 7 up-regulated and 11 down-regulated DETFs involved in FLT1 regulation were discovered. Furthermore, JASPAR (Rauluseviciute *et al.*, 2024) was used to determine whether the discovered DETFs had binding sites present at the promoter of the *FLT1* gene. Collectively, nine DETFs, including EPAS1, ETS1, TBX3, CEBPB, FLI1, TEAD4, GATA4, TBX2, and LMX1B were captured (Table 2).

**Table 2.** Transcription factors enrichment analysis using the ChEA3 tool.

Gene name	Log2FC	Mean rank	Overlapping genes
**EPAS1**	2.41	72.8	336
**ETS1**	−1.34	105.7	633
**TBX3**	1.83	139	352
**CEBPB**	2.52	169	638
**FLI1**	−2.65	184.3	995
**TEAD4**	−3.38	252.3	638
**GATA4**	−2.09	324.2	569
**TBX2**	−3.51	333	188
**LMX1B**	−1.93	477	166

The prediction is the result obtained by integrating multiple databases. Different databases have different rankings, so the higher the mean rank is, the more accurate it will be.

To gain more insights into DETFs, we also performed TF enrichment analysis with another tool ‘KnockTF’ (Feng *et al.*, 2024). Eleven up-regulated and 14 down-regulated DETFs associated with FLT1 regulation were detected. After confirmation of binding sites using JASPAR, we obtained nine significant DETFs: FLI1, TEAD4, ARNT, CEBPB, FOXM1, ERF, PRDM1, TFAP2A, and NR2F2 (Table 3).

**Table 3.** Transcription factors enrichment analysis using the KnockTF tool.

Gene name	Log2FC	Number of intersecting genes	*P* value
**FLI1**	−2.65	1064	4.18E−29
**TEAD4**	−3.38	829	7.62E−16
**ARNT**	1.15	1056	4.54E−15
**CEBPB**	2.52	661	6.97E−12
**FOXM1**	−1.75	1691	7.62E−16
**ERF**	−1.16	810	1.77E−06
**PRDM1**	−2.40	485	0.000163
**TFAP2A**	1.34	748	0.000201
**NR2F2**	−1.55	1214	0.00321

### Validation of DETFs at the mRNA level

**Figure 2.**
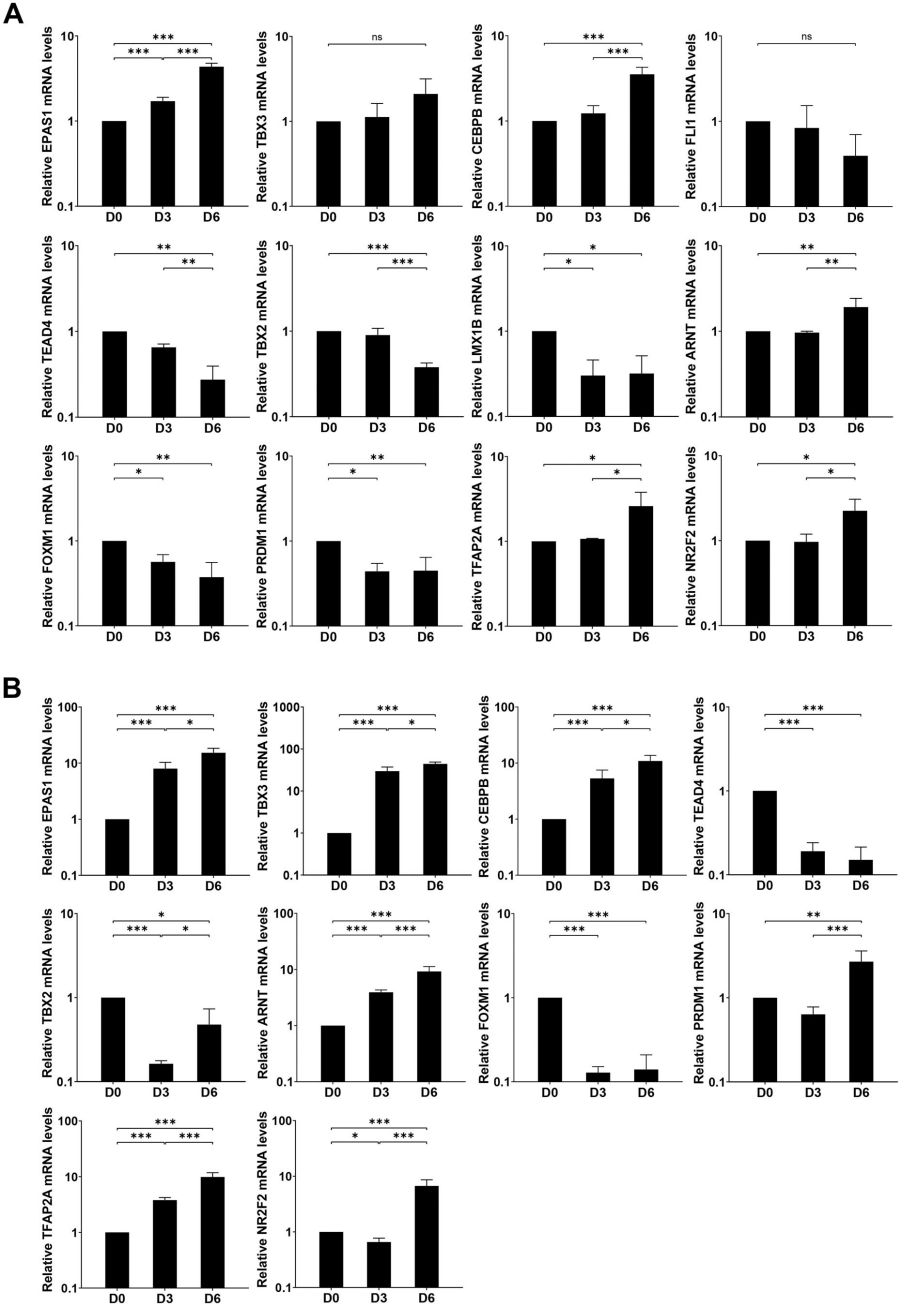
**Messenger RNA expression of transcription factors during differentiation from cytotrophoblasts.** Cytotrophoblasts (CTBs) were cultured with extravillous trophoblast (EVT) or syncytiotrophoblast (STB) medium for 6 days, qPCR was used to measure the expression of transcription factors (TFs) on Day 0 (undifferentiated CTBs), and Days 3 and 6 (both differentiated EVTs (**A**) or STBs (**B**)). Data were expressed relative to CTBs (Day 0). Results were normalized for the housekeeping genes *GAPDH*, *TBP*, and *HPRT*. Data are presented as means ± SD from three independent experiments. One-way ANOVA was performed on log-transformed gene expression. *P <* *0.05, **P <* *0.01, ***P <* *0.001.

To verify the mRNA levels of DETFs identified with RNA sequencing, quantitative PCR (qPCR) was performed to confirm the differential mRNA expression of TFs on Day 0 (undifferentiated CTBs) and 3 and 6 days of EVT differentiation. ETS1, ERF, and GATA4 were undetectable in EVTs. Compared to CTBs, EPAS1, CEBPB, ARNT, and TFAP2A increased, and TEAD4, TBX2, LMX1B, FOXM1, and PRDM1 decreased significantly in expression in EVTs (Day 6), which was consistent with the RNA sequencing results. There was no remarkable difference for TBX3 and FLI1 mRNA expression between CTBs and EVTs. The mRNA level of NR2F2 was increased significantly in EVTs (Day 6) compared to CTBs (Fig. 2A).

Since sFLT1 is also up-regulated in STBs, we investigated the mRNA expression of DETFs during STB differentiation from CTBs (Fig. 2B). We detected 10 DETFs, of which 7 DETFs (EPAS1, TBX3, CEBPB, ARNT, PRDM1, TFAP2A, and NR2F2) increased significantly at the mRNA level upon CTB differentiation into STBs. The expression of TEAD4 and FOXM1 decreased in STBs compared to CTBs. TBX2 mRNA expression decreased on Day 3 (STBs) compared to Day 0 (CTBs) but increased significantly on Day 6 (STBs) when compared to Day 3 (STBs). Collectively, the seven DETFs (EPAS1, CEBPB, ARNT, TFAP2A, NR2F2, TEAD4, and FOXM1), whose expression was altered during STB differentiation, overlapped with those identified in EVT differentiation.

### Validation of DETFs and sFLT1/FLT1 at the protein level

**Figure 3.**
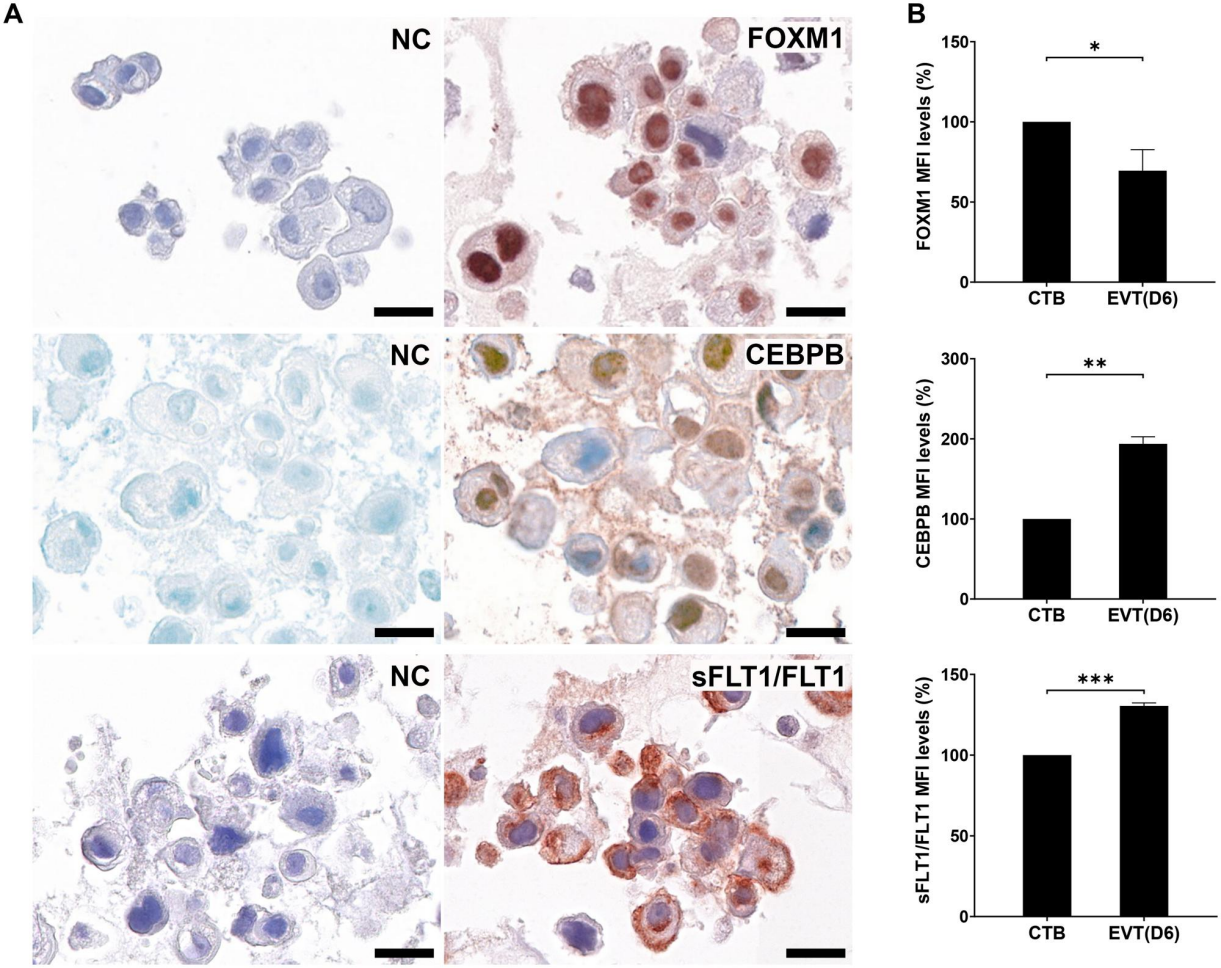
**Protein expression of the transcription factors FOXM1 and CEBPB, and sFLT1/FLT1.** Cytotrophoblasts (CTBs) were cultured with extravillous trophoblast (EVT) medium for 6 days; immunohistochemistry was performed to study the localization of FOXM1, CEBPB, and sFLT1/FLT1. (**A**) Representative images of FOXM1 staining (upper panel), CEBPB staining (middle panel), and sFLT1/FLT1 (lower panel). Negative mouse or rabbit control antibodies were used (NC). FOXM1 and CEBPB were concentrated in the nucleus, and sFLT1/FLT1 were in the cell membrane and cytoplasm. The scale bars represent 20 µm. (**B**) Flow cytometry was used to quantify the protein expressions of FOXM1, CEBPB, and sFLT1/FLT1 on Day 0 (undifferentiated CTBs) and Day 6 (differentiated EVTs). Data are expressed relative to CTBs (Day 0). Data are presented as means ± SD from three independent experiments. A paired *t*-test was performed on log-transformed gene expressions. *P <* *0.05, **P <* *0.01.

Based on the association of FOXM1 with angiogenesis in early placentation (Monteiro *et al.*, 2019) and the known immune-related role of CEBPB in early pregnancy (Chang *et al.*, 2023), we selected these two factors for further investigation. We first performed immunochemistry of FOXM1 and CEBPB upon CTB differentiation into EVTs and observed intensive nuclear staining of both FOXM1 and CEBPB (Fig. 3A). Then, we used flow cytometry to quantify the protein levels of FOXM1 and CEBPB during differentiation. The protein expression of FOXM1 decreased significantly in EVTs (Day 6) when compared to CTBs, and CEBPB significantly increased in protein expression (Fig. 3B). Representative analysis of the flow cytometry is shown in Supplementary Fig. S1.

Next, we validated the protein expression of sFLT1/FLT1 upon CTB differentiation into EVTs using immunochemistry and flow cytometry. As anticipated, FLT1 localized to the cell membrane and sFLT1/FLT1 in the cytoplasm (Fig. 3A), and sFLT1/FLT1 was markedly elevated in EVTs compared to CTBs (Fig. 3B).

### Verification of the regulated effect of DETFs on sFLT1 expression

**Figure 4.**
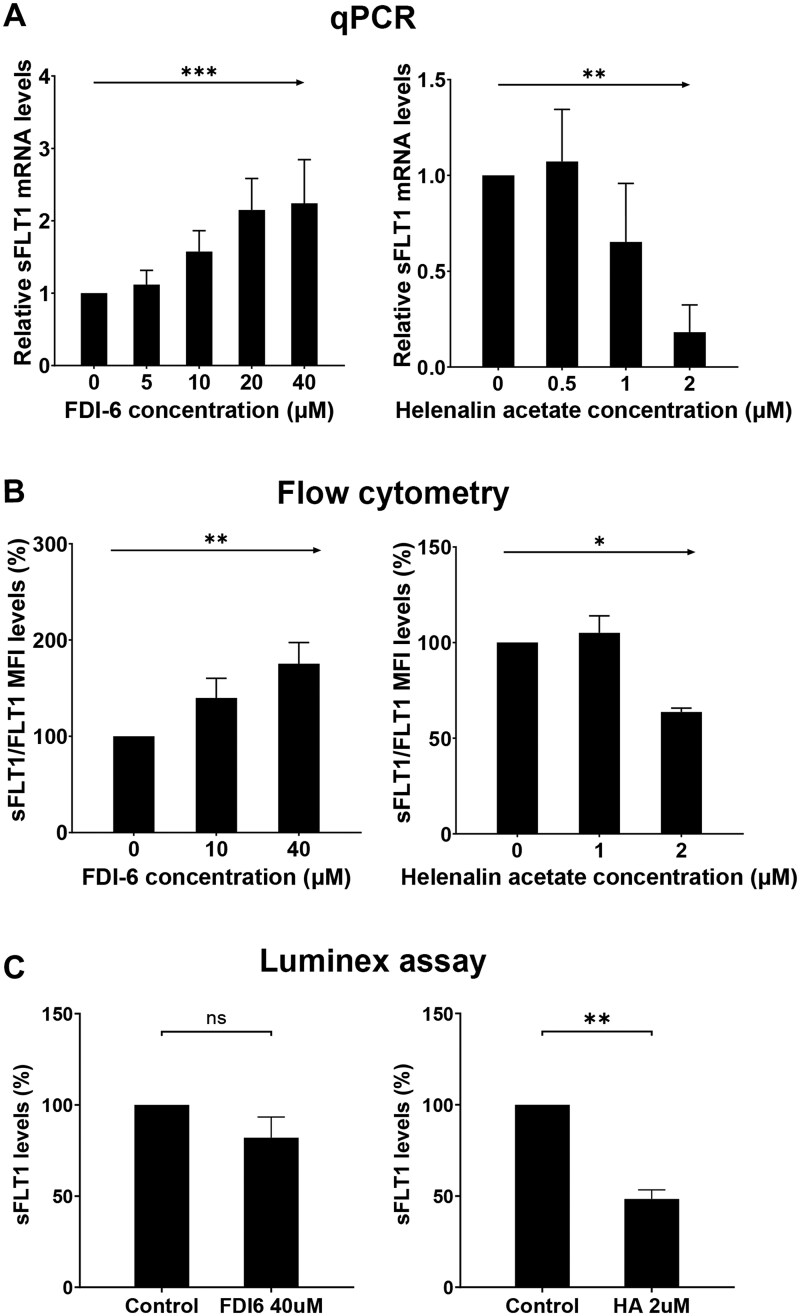
**The regulatory effect of transcription factors on sFLT1 expression in trophoblasts.** Cytotrophoblasts (CTBs) were incubated with 5, 10, 20, and 40 µM of FOXM1 inhibitor (FDI-6) for 24 h (predicted as transcription repressor). To investigate the transcription activation of CEBPB on sFLT1, CTBs were differentiated into extravillous trophoblasts (EVTs) for 3 days. Different concentrations of a CEBPB inhibitor (helenalin acetate, 0.5, 1, and 2 µM) were added to EVT medium for 48 h. As a control, cells were cultured in CTB or EVT medium without transcription factor (TFs) inhibitors. After that, sFLT1 mRNA and sFLT1/FLT1 protein expression were measured using qPCR and flow cytometry. A Luminex assay was used to detect the secreted sFLT1 level in the culture medium. Soluble FLT1 mRNA levels were normalized to the housekeeping genes *GAPDH*, *TBP*, and *HPRT*. Data are expressed relative to their own control without inhibitor. (**A**) Soluble FLT1 mRNA levels and (**B**) sFLT1/FLT1 protein expressions in trophoblasts, determined by flow cytometry, with an increasing dose of FDI-6 or helenalin acetate. (**C**) Soluble FLT1 levels in trophoblast culture medium, assessed by Luminex analysis, with 40 µM FDI-6 or 2 µM helenalin acetate treatment. Linear regression analysis was performed on log-transformed concentration of TFs and sFLT1 mRNA or sFLT1/FLT1 protein expression (A and B). A paired *t*-test was performed on log-transformed secreted sFLT1 levels (C). *P <* *0.05, **P <* *0.01, ***P <* *0.001.

To establish the effect of FOXM1 on sFLT1 expression (predicted as a transcription repressor), we cultured CTBs with an increasing dose of the FOXM1 inhibitor FDI-6 (5, 10, 20, 40 µM). The mRNA level of sFLT1 increased significantly and in a dose-dependent manner (Fig. 4A, *P *<* *0.001). Consistently, flow cytometry revealed a gradual increase in sFLT1/FLT1 protein levels in response to increasing doses of FDI-6 (Fig. 4B, *P *<* *0.01). Representative analysis of the flow cytometry is shown in Supplementary Fig. S2.

We also investigated whether a CEBPB inhibitor could downregulate sFLT1 expression. CTBs were incubated in EVT medium for 3 days and then stimulated with different concentrations of the CEBPB inhibitor helenalin acetate (0.5, 1, 2 µM). Soluble FLT1 mRNA expression decreased significantly and in a dose-dependent manner with increasing concentrations of helenalin acetate (Fig. 4A, *P* < 0.01). Similarly, a significant reduction in sFLT1/FLT1 protein levels was observed following increasing doses of helenalin acetate (Fig. 4B, *P *<* *0.05). Representative flow cytometry analysis is shown in Supplementary Fig. S2.

Finally, we investigated the secreted sFLT1 levels in trophoblast culture medium without or with TF inhibitors using the Luminex assay. There was no significant difference of sFLT1 level in medium without or with FDI-6 (40 µM). However, in the medium with helenalin acetate (2 µM), sFLT1 levels were remarkably reduced compared to the medium without CEBPB inhibitor (Fig. 4C, *P *<* *0.01).

### DETFs expression in first-trimester placentas

**Figure 5.**
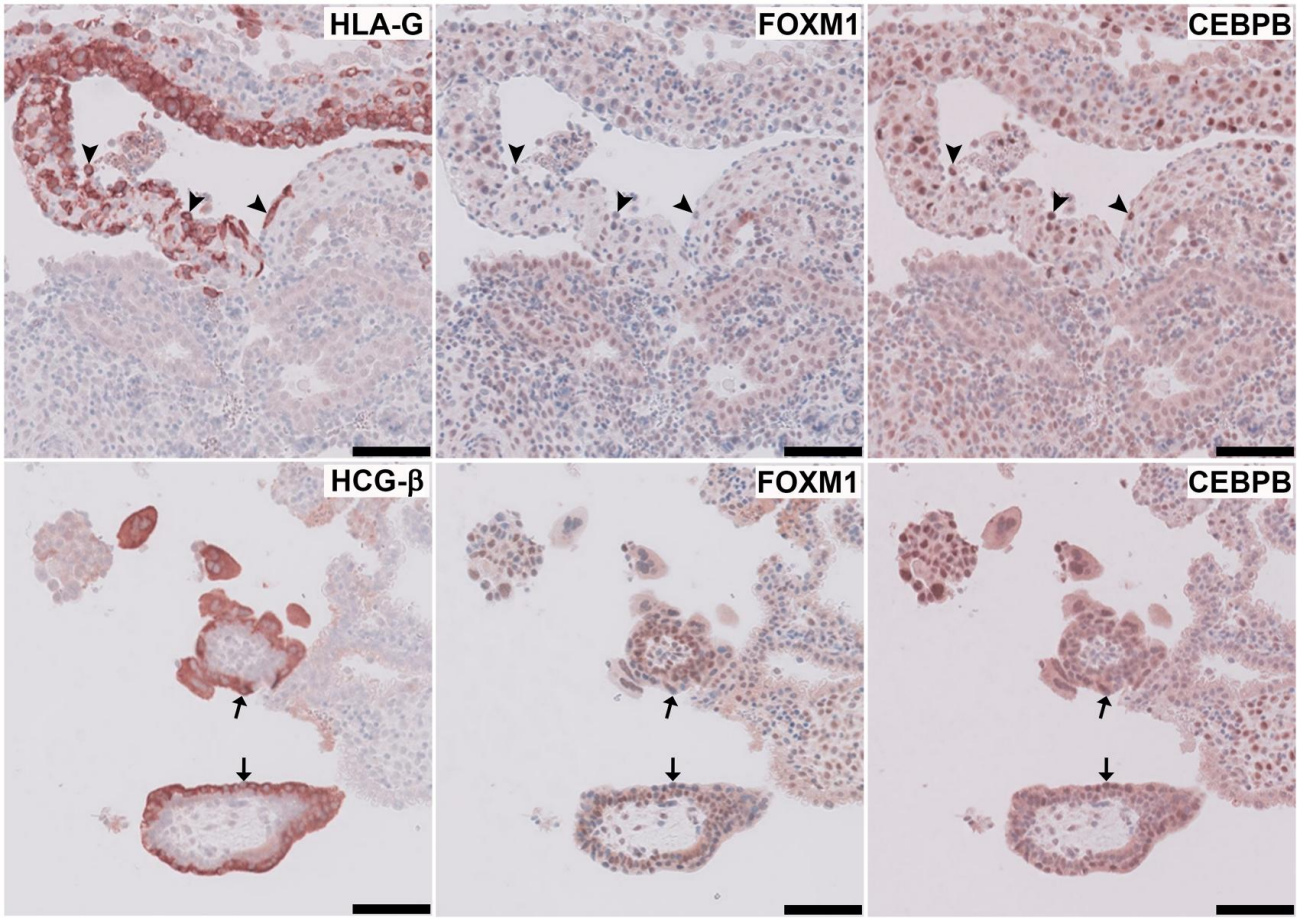
**Expression of transcription factors in first-trimester placentas.** HLA-G and HCG-β were stained as markers for extravillous trophoblasts (EVTs) and syncytiotrophoblasts (STBs), respectively. Representative images of FOXM1 and CEBPB staining in placental tissue from first trimester. In both EVTs (arrowheads, upper panel) and STBs (arrows, lower panel), CEBPB was highly present and the expression of FOXM1 was low. The scale bars represent 100 µm.

As we found that FOXM and CEBPB could regulate sFLT1 expression, we next validated the protein pattern of FOXM1 and CEBPB in human tissue. For this, we performed immunochemistry of FOXM1 and CEBPB on three first-trimester placentas. HLA-G and HCG-β were stained as markers of EVTs and STBs, respectively. In all three placental tissues from first trimester, CEBPB was highly present in EVTs and STBs, while the expression of FOXM1 in EVTs and STBs was lower compared to that in CTBs. Representative images of three placental stainings are shown in Fig. 5.

### Signal pathway enrichment analysis related to FLT1 and PPI analysis

**Figure 6.**
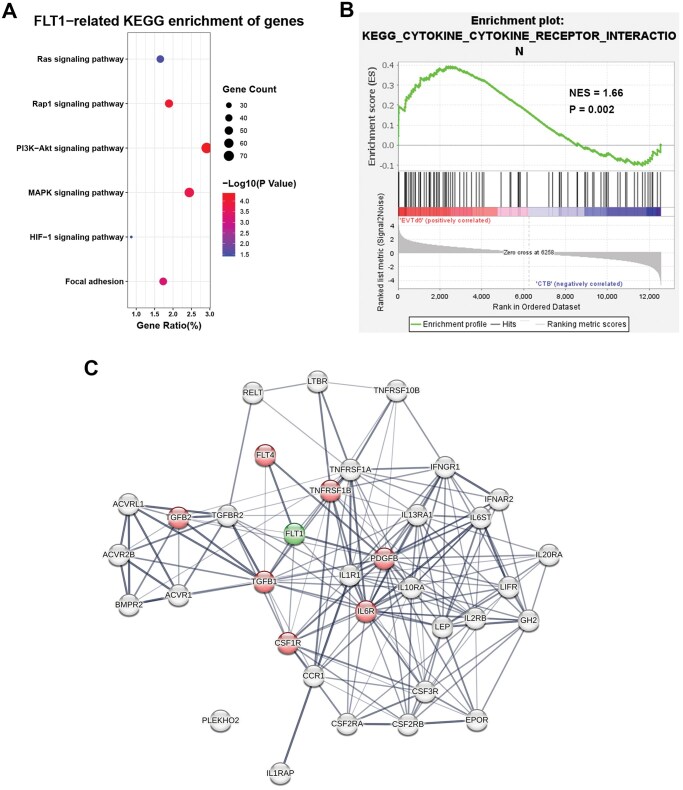
**Pathway analysis of differentially expressed genes between extravillous trophoblasts (EVTs) and cytotrophoblasts (CTBs).** Kyoto encyclopedia of genes and genomes (KEGG) pathway enrichment analysis of DEGs in group EVTd6 versus CTB. (**A**) Bubble charts illustrating the significant pathways where FLT1 is involved. The size of the dots represents the number of enriched genes, and the shades of color represent *P* values. Gene ratio = count/set size. (**B**) Gene set enrichment analysis (GSEA) snapshot of significant FLT-related enrichment. (**C**) Protein–protein interaction network of core genes obtained from GSEA. Red bubbles represent genes that interacted with FLT1. Line thickness indicates the strength of data support. DEGs, differentially expressed genes; NES, normalized enrichment score.

To explore the potential function of FLT1 (including both full-length receptor and its soluble isoforms) during EVTs differentiation from CTBs, we executed KEGG enrichment analysis on DEGs identified from groups EVTd6 and CTB, using DAVID 2021 software (Sherman *et al.*, 2022). The FLT1-related pathways were collected and visualized by bubble plots (Fig. 6A). The main biological pathways included the P13K–Akt signaling pathway, the Rap1 signaling pathway, the MAPK signaling pathway, Focal adhesion, the Ras signaling pathway, and the HIF-1 signaling pathway.

To better study the signaling pathways that are associated with FLT1, we used all detected genes from groups EVTd6 and CTB and carried out gene set enrichment analysis (Subramanian *et al.*, 2005). Key pathways and core-related genes were gained. The only significant enrichment wherein FLT1 was involved was ‘cytokine–cytokine receptor interaction’ (FDR = 0.052, *P =* 0.002). A snapshot of the enrichment analysis is shown in Fig. 6B.

To further understand the potential immunomodulatory role of FLT1, we performed PPI analysis on STRING online software (Szklarczyk *et al.*, 2023) using all the core genes enriched in the cytokine receptor interaction pathway. The genes that interacted with FLT1 were FLT4, PDGFB, TGFB1, IL6R, TNFRSF1B, CSF1R, and TGFB2 (Fig. 6C).

## Discussion

Here, we show that the expression profile of genes, including TFs, is highly altered when CTBs differentiate to EVTs. Moreover, we identified 197 differentially expressed TFs, of which 15 were predicted to regulate sFLT1 expression. We validated the mRNA levels of the predicted TFs with qPCR and confirmed that 10 out of 15 were differentially expressed during differentiation. We also showed that inhibitors for the TFs FOXM1 and CEBPB affect sFLT1 expression *in vitro*. We verified FOXM1 and CEBPB protein expression upon CTB differentiation into EVTs and in first-trimester placentas. Finally, we identified pathways in which FLT1 is involved during EVT differentiation.

Combining two TF enrichment analysis tools (ChEA3 and KnockTF), we obtained a list of TFs that are likely to regulate FLT1 expression. After further confirmation of binding sites at the promotor of the *FLT1* gene, 15 DETFs were identified. We further performed qPCR to validate DETF levels upon CTB differentiation into EVTs, and into STBs, where we previously found (Yong *et al.*, 2023) that sFLT1 is also up-regulated. Interestingly, most of the DETFs identified during EVT differentiation exhibit a similar expression pattern during STB differentiation, despite the diversity of these two differentiation pathways. This similarity suggests a common regulatory mechanism, where sFLT1 represents the connecting link influenced by these TFs.

Among the predicted DETFs, some are known to play a role in placental development. For instance, FOXM1 is an important regulatory factor in trophoblast proliferation, migration, and invasion (Xie *et al.*, 2015; Mao *et al.*, 2021; Penailillo *et al.*, 2024). Monteiro *et al.* (2019) observed that FOXM1 engages in early placentation events and is associated with angiogenesis. However, whether FOXM1 is involved in sFLT1 regulation in the early stage of pregnancy is unclear. We treated CTBs with a FOXM1 inhibitor and found that FOXM1 significantly down-regulated the expression of sFLT1 in cells. Coincidentally, studies in term placentas showed that placental FOXM1 at term is down-regulated in PE where sFLT1 is overexpressed (Cui *et al.*, 2018; Monteiro *et al.*, 2019). Collectively, our finding that FOXM1 acts as a transcriptional repressor and regulates sFLT1 expression unravels a possible novel mechanism of PE and may provide a target for treatment. Although we observed alterations of sFLT1 mRNA and sFLT1/FLT1 protein expression in cells treated with the FOXM1 inhibitor, no corresponding changes were detected in secreted sFLT1 levels in the culture medium. A reason for this could be the relatively short incubation time of the FOXM1 inhibitor compared to that of the other TF inhibitor employed in our study, and the difference was below the detection threshold of the Luminex assay. It is also possible that sFLT1 protein levels remain unchanged, as flow cytometry detected both membrane-bound FLT1 and sFLT1, whereas the Luminex assay most likely specifically quantified sFLT1.

The essential role of CEBPB on uterine stromal cell decidualization has been demonstrated in both human and mice (Li *et al.*, 2015; Huang *et al.*, 2022). Interestingly, decidual cells from preeclamptic women exhibit defective decidualization and overexpress sFLT1 (Sahu *et al.*, 2019). Similarly, Cottrell *et al.* (2017) observed that sFLT1 expression negatively coincides with decidualization of human endometrial stromal cells. Taken together, there is a strong correlation between abnormal decidualization, where CEBPB is a mediator, and excessive sFLT1, a phenomenon that is associated with PE. To verify whether CEBPB could regulate the expression of sFLT1 *in vitro*, we added a CEBPB inhibitor during EVT differentiation and observed that CEBPB upregulates sFLT1 expression significantly. We showed for the first time that CEBPB regulates sFLT1 expression in trophoblasts. It may mean that this control of sFLT1 expression levels is a requirement for maintaining successful decidualization. Considering the potential cytotoxic effect of the CEBPB inhibitor (helenalin acetate), we performed a cell viability assay with different concentrations of helenalin acetate (up to 4 µM) on CTBs and did not observe a significant cytotoxic effect. However, it would still be necessary to take the side effects into account if applying a higher concentration of helenalin acetate for treatment.

Furthermore, TFAP2A is not only shown to mediate decidualization in stromal cells during pregnancy but also affects sFLT1 expression *in vitro* (Manley *et al.*, 2020). This confirms our prediction of the regulatory effect of TFAP2A on sFLT1 expression and suggests that the software algorithm for predicting TFs is reliable and can be used in future investigations.

Several studies have shown that sFLT1 has anti-inflammatory properties in inflammation-associated diseases, such as psoriasis and Type 1 diabetes (Schonthaler *et al.*, 2009; Bus *et al.*, 2017). We previously reported (Yong *et al.*, 2023) that sFLT1 is highly present in EVTs and STBs from first-trimester placentas. Both EVTs and STBs have been extensively described in the literature as being involved in immune tolerance (Xu *et al.*, 2021), which is crucial for protecting the fetus carrying paternal antigens from the maternal immune system to ensure successful pregnancy (Fuhler, 2020). Therefore, we hypothesized that increased sFLT1 contributes to immune regulation at the maternal–fetal interface. Here, we found that down-regulated FOXM1 and up-regulated CEBPB (both at the mRNA level and protein pattern) are not only seen in EVTs but also in STBs. In addition, the regulatory effects of CEBPB and FOXM1 on sFLT1 expression are shown during EVT differentiation. Increased evidence suggests that FOXM1 and CEBPB are closely related to immune regulation. For instance, FOXM1 is expressed by various immune cells, including T cells, B cells, monocytes, macrophages, and dendritic cells, and it mediates the development of immune cells (Zheng *et al.*, 2023). Meanwhile, CEBPB has been described to regulate the expression of genes involved in immune responses (Kinoshita *et al.*, 1992; Roy *et al.*, 2002). Moreover, a study by Zhu *et al.* (2023) showed that aberrant degradation of CEBPB inhibits macrophage Type 2 (M2) polarization in atopic dermatitis skin. Similarly, the importance of CEBPB on M2-like polarization of macrophages in the early decidua during normal pregnancy was confirmed in a PIGF knockout mouse model (Chang *et al.*, 2023). Collectively, the alterations in FOXM1 and CEBPB levels in both EVTs and STBs, and their regulatory effect on sFLT1 expression, support the involvement of sFLT1 in the immunomodulatory effect at the maternal–fetal interface. However, it is still possible that the regulatory effect of FOXM1 and CEBPB on sFLT1 expression is independent of the immunomodulatory roles of these TFs. More evidence is needed to confirm the anti-inflammatory properties of sFLT1 in early normal pregnancy and PE.

To investigate the role of sFLT1 during CTB differentiation into EVTs, we performed pathway analyses and PPI analysis. Interestingly, we found pathways that link FLT1 to components of the immune system. This finding further supports the notion that sFLT1 contributes to immune regulation at the maternal–fetal interface. Further investigation into the precise inflammatory effects of sFLT1 in pregnancy and PE needs to be done.

A limitation of our study is that we did not validate all predicted TFs to confirm their regulatory effects on sFLT1 expression. Moreover, to thoroughly validate the interactions between TFs and the promotor region of sFLT1, advanced approaches such as electrophoretic mobility shift assay (EMSA), chromatin immunoprecipitation, and reporter gene assays will be required.

In conclusion, we identified TFs FOXM1 and CEBPB that could regulate placental sFLT1 expression, at least at the mRNA level, and provided support that sFLT1 has immunomodulatory effects in early pregnancy. These observations may help to eventually provide therapeutic targets in PE. When considering the treatment of PE, it is crucial to account for the possible negative consequences of removing sFLT1 and to maintain sFLT1 at a physiological level.

## Supplementary Material

gaaf031_Supplementary_Data

## Data Availability

The sequencing data supporting the findings of this study are openly available in the GEO repository. The GEO accession number is GSE294617.
