# Supplementary material for: Identification of transcription factors that regulate placental sFLT1 expression
Source: Mol Hum Reprod. 2025 Jul 9;31(3):gaaf031. doi: 10.1093/molehr/gaaf031 (PMC12286775; doi:10.1093/molehr/gaaf031)
Supplement: gaaf031_Supplementary_Data [file gaaf031_supplementary_data.zip › Supplementary information revised.pdf]

Identification of transcription factors that regulate placental sFLT1 expression

Q Yong, C van der Keur, J D H Anholts, H Kapsenberg, H Mei, J A Bruijn, M Eikmans, H J Baelde.

Supplementary Figure S1: Protein expression of transcription factors.

Supplementary Figure S2: The regulatory effect of transcription factors on sFLT1 expression in trophoblasts.

Supplementary Table S1: Differentially expressed genes in group EVT d3 versus CTB. (Table provided as a separate file)

Supplementary Table S2: Differentially expressed genes in group EVT d6 versus CTB. (Table provided as a separate file)

Supplementary Table S3: Differentially expressed genes in group EVT d6 versus EVT d3. (Table provided as a separate file)

Supplementary Table S4: Differentially expressed transcription factors in group EVT d6 versus CTB. (Table provided as a separate file)

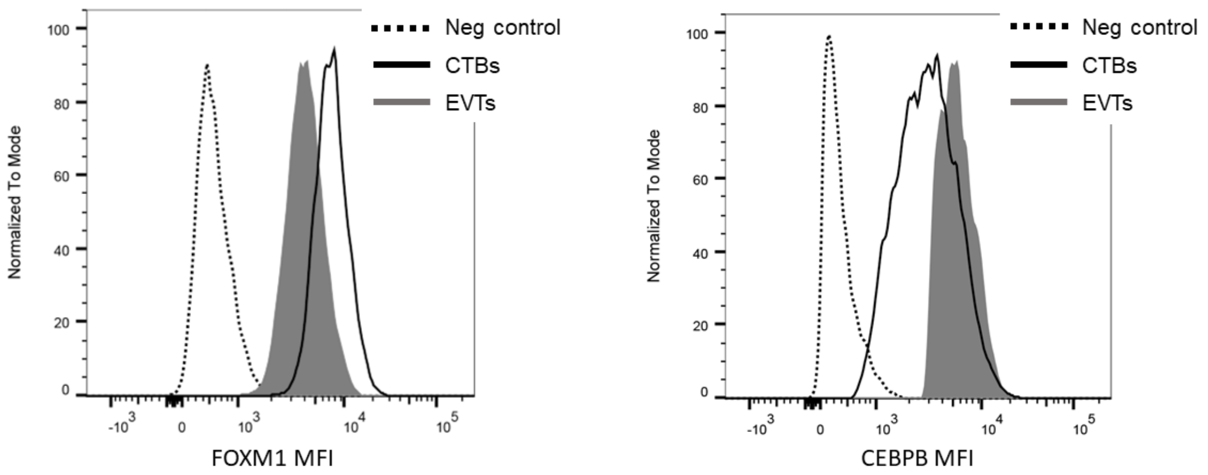

Supplementary Figure S1: Protein expression of transcription factors. Representative picture of the flow cytometry analysis. CTBs were cultured with EVT medium for 6 days, flow cytometry was used to quantify the protein expressions of FOXM1 and CEBPB on day 0 (undifferentiated CTBs) and day 6 (differentiated EVTs). CTBs, cytotrophoblasts; EVTs, extravillous trophoblasts; Neg control, negative control; MFI, medium fluorescence intensity.

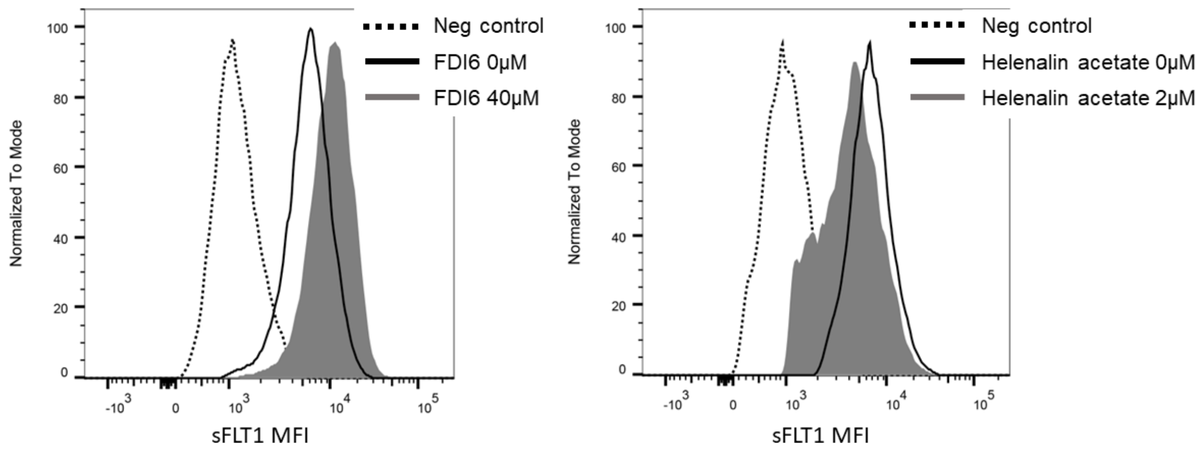

Supplementary Figure S2: The regulatory effect of transcription factor inhibition on sFLT1 expression in trophoblasts. Representative picture of the flow cytometry analysis. CTBs were incubated with 10 µM or 40 µM FOXM1 inhibitor (FDI-6) for 24h or differentiated into EVTs for 3 days following by adding 1 µM or 2 µM CEBPB inhibitor (Helenalin acetate). As a control, cells were cultured in CTB or EVT medium without TFs inhibitors. After that, protein expressions were measured using flow cytometry. CTBs, cytotrophoblasts; EVTs, extravillous trophoblasts; TFs, transcription factors; Neg control, negative control; MFI, medium fluorescence intensity.
